# A Quantum Proxy Signature Scheme Without Restrictions on the Identity and Number of Verifiers

**DOI:** 10.3390/e27111171

**Published:** 2025-11-19

**Authors:** Siyu Xiong

**Affiliations:** Discipline Construction Office, Civil Aviation Flight University of China, Guanghan 618307, China; siyuxiong1996@outlook.com

**Keywords:** quantum digital signature, quantum proxy signature, quantum Fourier transform, quantum cryptography

## Abstract

Quantum digital signatures (QDS) establish a framework for information-theoretically secure authentication in quantum networks. As a specialized extension of QDS, quantum proxy signatures facilitate secure delegation of signing privileges in distributed quantum environments. However, existing schemes require the predefinition of verifier identities at the system setup phase, which fundamentally constrains their deployment in real-world scenarios. To address this constraint, we propose a quantum proxy signature scheme supporting verification by arbitrary parties without pre-registration while maintaining information-theoretic security guarantees. This work presents a constructive approach to mitigating verification constraints in quantum proxy signature architectures.

## 1. Introduction

Digital signatures, a core modern cryptography technology, underpin data security and the fundamental trust framework for critical digital infrastructures in the digital era. Widely deployed public-key cryptosystems in current classical computing scenarios—typified by RSA [1] and ECDSA [2]—anchor their security on computational complexity-theoretic assumptions, with particular reliance on the intractability of the large integer factorization problem and the discrete logarithm problem in finite fields. The advent of quantum algorithm [3,4,5,6] has fundamentally undermined this foundation by solving classically hard computational problems in polynomial time, thereby compromising the security of current cryptographic infrastructures in the era of quantum computation.

The threat posed by quantum computing has catalyzed two distinct cryptographic paradigms: post-quantum cryptography [7,8,9,10,11], which develops classical algorithms resistant to quantum attacks, and quantum cryptography [12,13,14,15,16], which leverages quantum mechanical principles to achieve information-theoretic security. Within quantum cryptography, quantum digital signatures (QDS) represent a class of protocols that harness quantum states to provide non-forgeable and non-repudiable authentication of digital messages, achieving information-theoretic security unattainable in classical cryptography. Following Gottesman and Chuang’s seminal theoretical framework [17], subsequent QDS schemes utilizing single photons [18], entangled states [19], and coherent states [20] have advanced rapidly from conception to experimental realization, including metropolitan-scale demonstrations [21] and measurement-device-independent protocols enhancing practical security [22]. Recently, Du et al. demonstrated the feasibility of chip-based quantum-dot single-photon sources [23], paving the way for large-scale deployment and integration with existing optical fiber infrastructure.

The inherent requirement for authorization delegation in distributed quantum environments has motivated the development of quantum proxy digital signatures (QPDS) as a specialized branch of quantum digital signatures. QPDS addresses this critical need by enabling a fundamental cryptographic operation: the secure transfer of signing privileges from an original signer to a designated proxy while preserving the information-theoretic security of the quantum signature framework. This delegation capability establishes the foundation for practical applications where distributed authority is essential, including collaborative quantum computing [24], quantum blockchain networks [25], and quantum internet of things ecosystems [26].

The research community has proposed various QPDS implementations encompassing diverse technical approaches. In 2008, building upon the foundational principles of classical threshold signature scheme [27], Yang et al. proposed a threshold proxy quantum group signature scheme incorporating threshold-shared verification [28]. The field of quantum proxy signatures has demonstrated remarkable diversification and evolutionary trends, giving rise to numerous variants designed to fulfill specific security and application requirements. For instance, quantum blind proxy signatures [29,30,31] leverage their distinctive blinding property to ensure signature validity while preserving message content privacy, offering viable solutions for scenarios such as electronic voting. Furthermore, branches including quantum multi-proxy signatures [32,33,34], threshold quantum proxy signatures [35,36], and quantum proxy group signatures [37,38,39] have substantially enriched this architecture by respectively addressing critical challenges in distributed collaboration. These diverse schemes exhibit tremendous potential for constructing future complex information security ecosystems.

Nevertheless, current schemes predominantly adhere to a permissioned verification paradigm, wherein the verification mechanism is structurally coupled with specific participant identities or predetermined verifier sets. This design imposes stringent constraints on the verification process: verifiers must belong to a predefined set or complete registration during the system initialization phase. Notably, while schemes based on threshold cryptography offer enhanced security guarantees, their verification mechanisms are inherently bound to a fixed-size group of verifiers, thus incapable of supporting parallel, independent verification by an arbitrary number of verifiers in open environments. In open environments, the identities and quantities of verifiers are highly dynamic and unpredictable, a requirement that the existing permissioned verification paradigm fails to accommodate. This contradiction underscores the central challenge confronting the QPDS field: how to transcend the limitations on verifier identity and quantity while maintaining reasonable cryptographic assumptions, thereby achieving universal verification capability.

Addressing this challenge, this paper proposes a novel quantum proxy signature scheme which constructs the one-way function based on the quantum Fourier transform. The scheme integrates quantum key distribution for secure key establishment and utilizes unitary transformations for quantum state manipulation in the signature process. Our scheme achieves universal verification, allowing an arbitrary number of verifiers of any identity to verify signatures without requiring pre-registration. This approach effectively overcomes the verification constraints in existing architectures while providing a practical solution for distributed quantum applications.

The organization of the rest of this paper is as follows. We begin with an introduction to the core of our scheme—the construction of the quantum Fourier transform-based one-way function—in Section 2. Section 3 then gives the complete description of our quantum proxy signature scheme. Following that, a security analysis is conducted in Section 4. Finally, we summarize our work in Section 5.

## 2. The Construction of Quantum One-Way Function

The security of our quantum proxy signature scheme is built upon the quantum one-way function constructed using the quantum Fourier transform (QFT). This approach leverages the inherent computational asymmetry of QFT to create an irreversible transformation that is secure against both classical and quantum attacks, forming the foundational component of our scheme. Here, we provide the detailed description of this construction.

Based on the QFT, the one-way function $f_{QFT}$ can map the classical bit string *M* of length *m* to the quantum state consisting of *n* quantum bits. Suppose the classical bit string *M* of length *m* can be expressed as

(1) $$M=\left(M_{0},M_{1},\ldots ,M_{m-1}\right),$$

where $M_{i}\in \left\{0,1\right\}$. And a quantum state consisting of *n* qubits is the vector in the complex vector space of dimension $2^{n}$, which can be expressed as

(2) $$|\psi \rangle =\sum_{x=0}^{2^{n}-1}\alpha_{x}|x\rangle ,$$

where $\alpha_{x}$ represents the complex amplitude, and |x〉 refers to the ground state. QFT is a linear transformation that maps the computational basis state |x〉 to the Fourier basis state $|y\rangle$. For *n* qubits, the definition of QFT is

(3) $$|x\rangle \to \frac{1}{\sqrt{2^{n}}}\sum_{y=0}^{2^{n}-1}e^{2\pi ixy/2^{n}}|y\rangle ,$$

where $\frac{1}{\sqrt{2^{n}}}$ is the normalization factor, and *y* is the index of the Fourier basis state, ranging from 0 to $2^{n}-1$. Meanwhile, QFT is a unitary transformation, which satisfies

(4) $$QFT^{†}QFT=I.$$

When constructing a quantum one-way function based on the QFT, first, the classical bit string *M* is encoded into an integer *x*, that is

(5) $$x=\sum_{i=0}^{m-1}M_{i}2^{i}.$$

Subsequently, the QFT is applied to the computational basis state |x〉, resulting in the output quantum state

(6) $$|\psi_{QFT}\rangle =|f_{QFT}\left(M\right)\rangle =\frac{1}{\sqrt{2^{n}}}\sum_{y=0}^{2^{n}-1}e^{2\pi i\left(\sum_{i=0}^{m-1}M_{i}2^{i}\right)y/2^{n}}|y\rangle$$

Here, *n* should be large enough to accommodate all possible values of *x*, that is

(7) $$n\geq \left\lceil \log_{2}\left(2^{m}\right)\right\rceil =m.$$

When calculating $|f_{QFT}\left(M\right)\rangle$, the QFT needs to be executed, and its complexity for simulation on a classical computer is $O\left(n^{2}\right)$. However, the inverse transformation, that is, reversing from $|f_{QFT}\left(M\right)\rangle$ to *x* on a classical computer is not feasible because the output of QFT is a highly complex quantum state, which cannot be directly measured to obtain *x*.

Quantum one-way function based on the QFT anchors its security in the physical nature of quantum state phase information, thereby providing information-theoretic security without relying on computational assumptions. It inherently resists quantum computing attacks and seamlessly integrates with quantum protocols, making it readily adaptable to broader quantum-safe security frameworks.

## 3. The Proposed Scheme

A quantum proxy signature scheme primarily enables a designated proxy signer to securely sign a message on behalf of the original signer, while ensuring the authenticity, integrity, and non-repudiation of the signed message under information-theoretic security. The proposed quantum proxy signature scheme involves three types of participants, as follows.

- Original signer: This participant has the original signing authority. They share the key with the proxy signatory through quantum key distribution and delegate the signing authority to the proxy signatory.
- Proxy signer: After this participant shares a key with the original signer through quantum key distribution, they sign on behalf of the original signer.
- Trusted center: An advanced trusted node, possessing control over the entire scheme.

Here, taking the case of the minimum number of participants for each identity, that is, one participant for each identity, we provide the detailed introduction to the quantum proxy signature scheme that is publicly verifiable. The entire signature scheme consists of four stages: the initialization stage, the authorization stage, the signature stage, and the verification stage. The protocol is executed through four sequential stages involving three participants: **original signer (OS)**, **proxy signer (PS)**, and **trusted center (TC)**. The complete workflow is formalized in the algorithms. The following provides detailed explanations for each stage.

### 3.1. The Initialization Stage

During this stage, the trusted center (TC) collaborates with the original signer Alice and the proxy signer Bob by executing a quantum key distribution protocol, generating three sets of keys of length 2n, namely $K_{AB}$, $K_{TA}$, $K_{TB}$. Let S={AB,TA,TB} represent the set of key types, and I={1,2,…,n} represent the index set. The three sets of keys can be expressed as

(8) $$K:S\times I\times \left\{1,2\right\}\to Z_{2},\;\;K\left(s,i,j\right)=\left\{\begin{array}{cc} a_{i}, & s=AB,j=1\\ b_{i}, & s=AB,j=2\\ c_{i}, & s=TA,j=1\\ d_{i}, & s=TA,j=2\\ e_{i}, & s=TB,j=1\\ f_{i}, & s=TB,j=2 \end{array}\right.,$$

where s∈S, i∈I, and j∈{1,2}. TC performs the quantum entanglement establishment process described between the original signer and the proxy signer. Each of the three parties simultaneously generates 2n entangled pairs. The entangled pairs between Alice and Bob can be expressed as

(9) $$ES:\left(C,|\Phi_{\mathrm{init}\;}\rangle \right)\to |\Phi_{\mathrm{final}\;}\rangle =\overset{2n}{\underset{i=1}{⨂}}|\phi_{i}\rangle =\overset{2n}{\underset{i=1}{⨂}}\frac{1}{\sqrt{2}}\left(|0_{a_{i}}0_{b_{i}}\rangle +|1_{a_{i}}1_{b_{i}}\rangle \right),$$

where *C* is the control information of TC as the advanced node during the entanglement establishment process, that is, the relevant measurement results. Through this entanglement establishment process, Alice and Bob generate 2n pairs of entangled particles. The particle sequences owned by Alice and Bob are respectively denoted as ${|\phi \rangle }_{a}$ and ${|\phi \rangle }_{b}$, which can be written as

(10) $$|\phi_{a}\rangle =\left\{|\phi_{a_{1}}\rangle ,|\phi_{a_{2}}\rangle ,\ldots ,|\phi_{a_{2n}}\rangle \right\},\;|\phi_{b}\rangle =\left\{|\phi_{b_{1}}\rangle ,|\phi_{b_{2}}\rangle ,\ldots ,|\phi_{b_{2n}}\rangle \right\}.$$

Furthermore, TC also needs to prepare *n* quantum states based on the keys $K_{TA}$ and $K_{TB}$, and the composite system becomes

(11) $$|\Psi_{T}\rangle =\overset{n}{\underset{i=1}{⨂}}|\psi_{c_{i},f_{i}}\rangle =\overset{n}{\underset{i=1}{⨂}}\frac{1}{\sqrt{2}}\left[\left(1-f_{i}\right)|c_{i}\rangle +f_{i}\left(|0\rangle +{\left(-1\right)}^{c_{i}}|1\rangle \right)\right].$$

TC uses pairs of entangled particles as the quantum channel, and the prepared $|\Psi_{T}\rangle$ is sent to the original signer Alice through teleportation. The message that the original signer Alice needs to sign is assumed to be

(12) $$M={\left(m_{i}\right)}_{i=1}^{n},\;m_{i}\in \left\{0,1\right\}.$$

TC calculates the exclusive OR (XOR) result

(13) $$g={\left(g_{i}\right)}_{i=1}^{n}={\left(m_{i}⨁c_{i}⨁e_{i}\right)}_{i=1}^{n}$$

based on the keys $K_{TA}$ and $K_{TB}$.

Using this XOR result, TC calculates and publishes its outcome based on the quantum one-way function, which can be expressed as

(14) $$|\psi_{\mathrm{QFT}}\left(g\right)\rangle =\frac{1}{\sqrt{2^{n}}}\sum_{y=0}^{2^{n}-1}e^{2\pi i\left(\sum_{i=1}^{n}g_{i}\cdot 2^{i-1}\right)y/2^{n}}|y\rangle .$$

The initialization stage establishes the foundational quantum resources and cryptographic keys required for the protocol. Algorithm 1 details the steps performed by the trusted center to set up the system.
**Algorithm 1:** Initialization Stage**Input**: Security parameter *n***Output**: Shared keys $K_{AB},K_{TA},K_{TB}$, entangled states $|\phi_{a}\rangle ,|\phi_{b}\rangle$, transformed state $|\Psi_{T}\rangle$, public QFT output $|\psi_{\mathrm{QFT}}\left(g\right)\rangle$**1 Key Distribution via QKD;****2**  $K_{AB}\leftarrow \mathrm{QKD}\left(OS,PS\right)$;**3**  $K_{TA}\leftarrow \mathrm{QKD}\left(TC,OS\right)$;**4**  $K_{TB}\leftarrow \mathrm{QKD}\left(TC,PS\right)$;**5 Entanglement Establishment;****6**  $\left(|\phi_{a}\rangle ,|\phi_{b}\rangle \right)\leftarrow \mathrm{EstablishEntanglement}\left(OS,PS,2n\right)$;**7 State Preparation and Teleportation;****8**  $|\Psi_{T}\rangle \leftarrow {⨂}_{i=1}^{n}\frac{1}{\sqrt{2}}\left[\left(1-f_{i}\right)|c_{i}\rangle +f_{i}\left(|0\rangle +{\left(-1\right)}^{c_{i}}|1\rangle \right)\right]$;**9**  $\mathrm{Teleport}(|\Psi_{T}\rangle ,OS)$;**10 Public Parameter Publication;****11**  $g\leftarrow {\left(g_{i}\right)}_{i=1}^{n}={\left(m_{i}⊕c_{i}⊕e_{i}\right)}_{i=1}^{n}$;**12**  $|\psi_{\mathrm{QFT}}\left(g\right)\rangle \leftarrow \frac{1}{\sqrt{2^{n}}}\sum_{y=0}^{2^{n}-1}e^{2\pi i\left(\sum_{i=1}^{n}g_{i}\cdot 2^{i-1}\right)y/2^{n}}|y\rangle$;**13**  $\mathrm{Publish}(|\psi_{\mathrm{QFT}}\left(g\right)\rangle )$;**14 return** $K_{AB},K_{TA},K_{TB},|\phi_{a}\rangle ,|\phi_{b}\rangle ,|\Psi_{T}\rangle ,|\psi_{\mathrm{QFT}}\left(g\right)\rangle$;

### 3.2. The Authorization Stage

The original signer Alice constructs a proxy certificate information sequence characterized by a quantum state sequence, which indicates the identity information of the proxy signer, the valid period of the authorization, and other specific constraints that may exist regarding the signature authority granted to Bob, and the sequence is denoted as

(15) $$|\Upsilon_{auth}\rangle =\left\{|\gamma_{auth}^{1}\rangle ,|\gamma_{auth}^{2}\rangle ,...,|\gamma_{auth}^{2n}\rangle \right\},\;|\gamma_{auth}^{i}\rangle \in \left\{|0\rangle ,|1\rangle \right\}.$$

According to this sequence, Alice measures the sequence $|\phi_{a}\rangle$ obtained through entanglement establishment, and the set of measurement operators is

(16) $$\begin{array}{c} \Pi_{Z}=\left\{\Pi_{Z0}=|0\rangle \langle 0|,\;\Pi_{Z1}=|1\rangle \langle 1|\right\},\\ \Pi_{X}=\left\{\Pi_{X+}=\frac{1}{2}(|0\rangle +\left|1\rangle )(\langle 0\right|+\langle 1|),\;\Pi_{X-}=\frac{1}{2}(|0\rangle -\left|1\rangle )(\langle 0\right|-\langle 1|)\right\} \end{array},$$

Suppose the characteristic function is

(17) $$\chi \left(|\gamma_{auth}^{i}\rangle \right)=\left\{\begin{array}{cc} 1, & \;\mathrm{if}\;|\gamma_{auth}^{i}\rangle =|0\rangle \\ 0, & \;\mathrm{else}\; \end{array}\right.,$$

then the resulting measurement can be expressed as

(18) $$MR_{A}=\overset{2n}{\underset{i=1}{⨂}}\left(\chi \left(|\gamma_{auth}^{i}\rangle \right)\frac{\Pi_{Zm_{i}}|\phi_{a_{i}}\rangle }{\sqrt{\langle \phi_{a_{i}}|\Pi_{Zm_{i}}|\phi_{a_{i}}\rangle }}+\left(1-\chi \left(|\gamma_{auth}^{i}\rangle \right)\right)\frac{\Pi_{Xm_{i}}|\phi_{a_{i}}\rangle }{\sqrt{\langle \phi_{a_{i}}|\Pi_{Xm_{i}}|\phi_{a_{i}}\rangle }}\right),$$

Here, $m_{i}$ represents the measurement result of the *i*-th particle. Subsequently, Alice transforms the particle sequence $|\Psi_{T}\rangle$ received through teleportation from TC using the key $K_{AB}$, and after the transformation, it becomes

(19) $$|\Psi_{TA}\rangle =\left(\overset{n}{\underset{i=1}{⨂}}U\left(a_{i}\right)V\left(b_{i}\right)\right)|\Psi_{T}\rangle ,$$

where $U\left(a_{i}\right)$ satisfies $U\left(0\right)=\left|0\rangle \langle 0\right|+|1\rangle \langle 1|,U\left(1\right)=i\sigma_{y}=\left|0\rangle \langle 1\right|-\left|1\rangle \langle 0\right|$ and $V\left(b_{i}\right)$ satisfies $V\left(0\right)=\left|0\rangle \langle 0\right|-|1\rangle \langle 1|,V\left(1\right)=H=\frac{1}{\sqrt{2}}(|0\rangle +\left|1\rangle )\langle 0\right|+\frac{1}{\sqrt{2}}(|0\rangle -\left|1\rangle )\langle 1\right|$. Alice encrypts the content generated by the above process using the key $K_{AB}$ to generate the message

(20) $$|S_{A}\rangle =E_{K_{AB}}\left(|\Upsilon_{auth}\rangle ,MR_{A},|\Psi_{TA}\rangle \right),$$

where the encryption of the classical bit sequence $MR_{A}$ uses the XOR operation of the key, while the encryption methods for quantum states $|\Upsilon_{auth}\rangle$ and $|\Psi_{TA}\rangle$ are as follows: Firstly, each binary bit in the key $K_{AB}$ is mapped to a quantum bit; for example, 0 is mapped to $|0\rangle$, 1 is mapped to $|1\rangle$, and then a controlled operation is performed on the quantum state, which is controlled by the key bit. The commonly used controlled operation is the controlled NOT gate. That is, if the key bit is 0, no operation is performed on the quantum state; if the key bit is 1, a quantum NOT gate operation is performed on the quantum state. After the encryption is completed, Alice sends the message $|S_{A}\rangle$ to the proxy signer Bob. Algorithm 2 formalizes this authorization process.
**Algorithm 2:** Authorization Stage
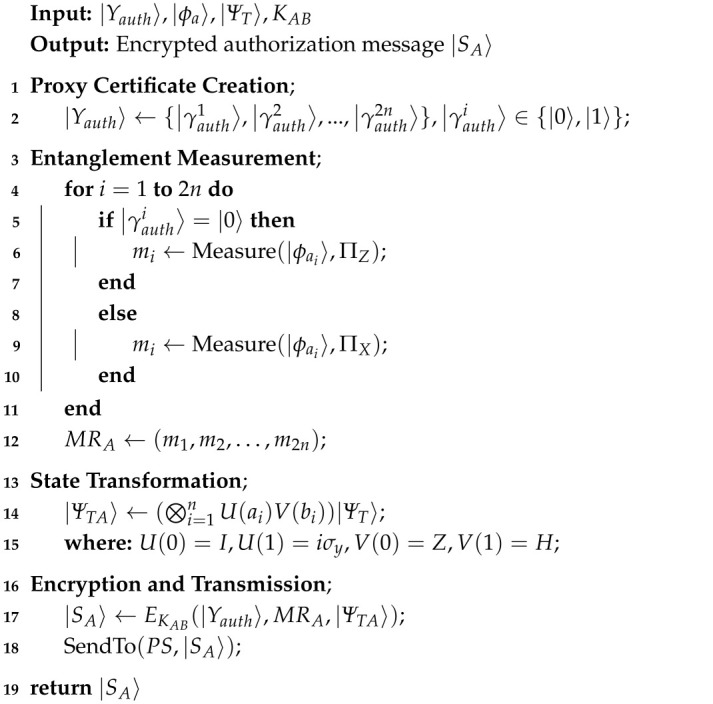


### 3.3. The Signature Stage

After receiving $|S_{A}\rangle$, the proxy signer Bob decrypts it using the key. The specific decryption method is the same as the encryption principle. Upon receiving $|\Upsilon_{auth}\rangle$, $MR_{A}$, and $|\Psi_{TA}\rangle$, Bob first verifies the authenticity and validity of the 2n-length quantum state sequence $|\Upsilon_{auth}\rangle$ before performing any further operations. Specifically, Bob conducts a verification procedure by measuring the received quantum state sequence $|\Upsilon_{auth}\rangle$ and comparing it with the locally held entangled state sequence $|\phi_{b}\rangle$ of identical length 2n, which was established during the prior entanglement distribution phase. If $|\gamma_{auth}^{i}\rangle =|0\rangle$, then the *Z* basis (i.e., {|0〉,|1〉}) is chosen to measure $|\varphi_{b_{i}}\rangle$; if $|\gamma_{auth}^{i}\rangle =|1\rangle$, then the *X* basis (i.e., {|+〉,|−〉}, where $|+\rangle =\left(|0\rangle +|1\rangle \right)/\sqrt{2}$ and $|-\rangle =\left(|0\rangle -|1\rangle \right)/\sqrt{2}$) is chosen to measure $|\varphi_{b_{i}}\rangle$. The measurement result is denoted as $MR_{B}$. If the condition

(21) $$MR_{A}=MR_{B}$$

is satisfied, the signature authorization is accepted; otherwise, the signature is rejected. If the signature authorization is accepted, then Bob will commence the subsequent proxy signature operation. First, Bob generates a sequence of 2n quantum random numbers

(22) $$R_{B}=\left(h_{1},j_{1},h_{2},j_{2},...,h_{n},j_{n}\right),\;h_{i},j_{i}\in \left\{0,1\right\}$$

as his private key. Bob performs the XOR operation with the generated key on the random number sequence between himself and TC, as well as between himself and Alice, and obtains the result

(23) $$K_{PB}=R_{B}⨁K_{TB}⨁K_{AB}=\left(p_{1},q_{1},p_{2},q_{2},...,p_{n},q_{n}\right).$$

Then Bob announces this sequence $K_{PB}$ as his public key. Bob uses the private key $R_{B}$ to transform the quantum state sequence $|\Psi_{TA}\rangle$ and generates the proxy signature as follows

(24) $$|S\rangle =\left(\overset{n}{\underset{i=1}{⨂}}\left(U\left(h_{i}\right)V\left(j_{i}\right)\right)\right)|\Psi_{TA}\rangle ,$$

where U(0)=|0〉〈0|+|1〉〈1|, $U\left(1\right)=i\sigma_{y}=\left|0\rangle \langle 1\right|-\left|1\rangle \langle 0\right|$, V(0)=|0〉〈0|−|1〉〈1|, $V\left(1\right)=H=1/\sqrt{2}(|0\rangle +\left|1\rangle )\langle 0\right|+1/\sqrt{2}(|0\rangle -\left|1\rangle )\langle 1\right|$. Algorithm 3 specifies the signature generation process.
**Algorithm 3:** Signature Stage
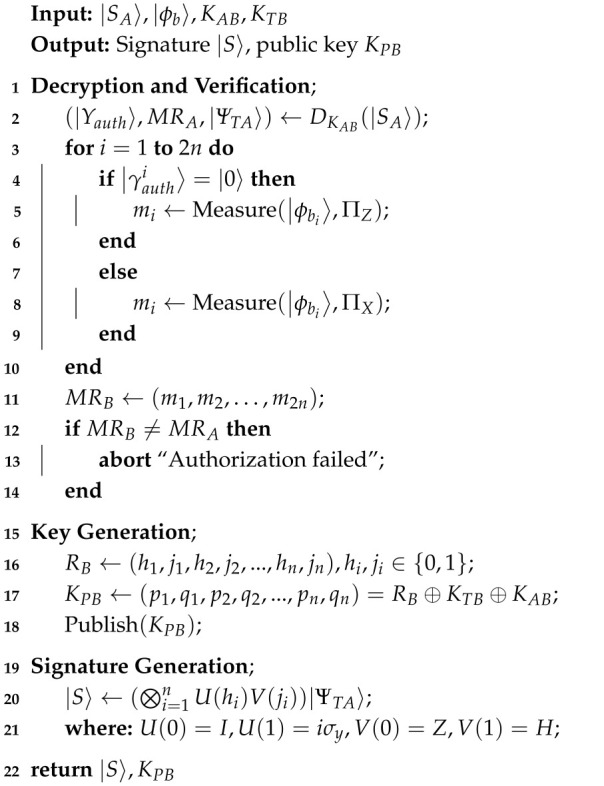


### 3.4. The Verification Stage

The generated proxy signature can be used for public verification, meaning that any user can verify the validity of the signature. Suppose user Charlie needs to verify the signature, then the following steps need to be executed. Charlie, using Bob’s public key $K_{PB}$, performs a transformation on the signature |S〉 to obtain the verification state

(25) $$|S_{C}\rangle =\left(\overset{n}{\underset{i=1}{⨂}}\left(U\left(p_{i}\right)V\left(q_{i}\right)\right)\right)|S\rangle ,$$

where U(0)=|0〉〈0|+|1〉〈1|, $U\left(1\right)=i\sigma_{y}=\left|0\rangle \langle 1\right|-\left|1\rangle \langle 0\right|$, V(0)=|0〉〈0|−|1〉〈1|, $V\left(1\right)=H=1/\sqrt{2}(|0\rangle +\left|1\rangle )\langle 0\right|+1/\sqrt{2}(|0\rangle -\left|1\rangle )\langle 1\right|$. Charlie measured the verification state using the Z basis measurement operator

(26) $$\Pi_{Z}=\left\{\Pi_{Z0}=|0\rangle \langle 0|,\Pi_{Z1}=|1\rangle \langle 1|\right\},$$

and the result was recorded as

(27) $$MR_{C}=\left(mr_{1},mr_{2},...,mr_{n}\right).$$

Charlie combines the measurement result with the message bit using an XOR operation, obtaining

(28) $$g_{C}=\left(g_{C}^{1},g_{C}^{2},...,g_{C}^{n}\right)=\left(mr_{1}⨁m_{1},mr_{2}⨁m_{2},...,mr_{n}⨁m_{n}\right).$$

After that Charlie calculated the result

(29) $$|\psi_{\mathrm{QFT}}\left(g_{C}\right)\rangle =\frac{1}{\sqrt{2^{n}}}\sum_{y=0}^{2^{n}-1}e^{2\pi i\left(\sum_{i=1}^{n}g_{C}^{i}\cdot 2^{i-1}\right)y/2^{n}}|y\rangle$$

based on the quantum Fourier transform. If

(30) $$|\psi_{\mathrm{QFT}}\left(g_{C}\right)\rangle =|\psi_{\mathrm{QFT}}\left(g\right)\rangle ,$$

the signature is valid; otherwise, the signature is rejected. Algorithm 4 defines the universal verification procedure.
**Algorithm 4:** Verification Stage
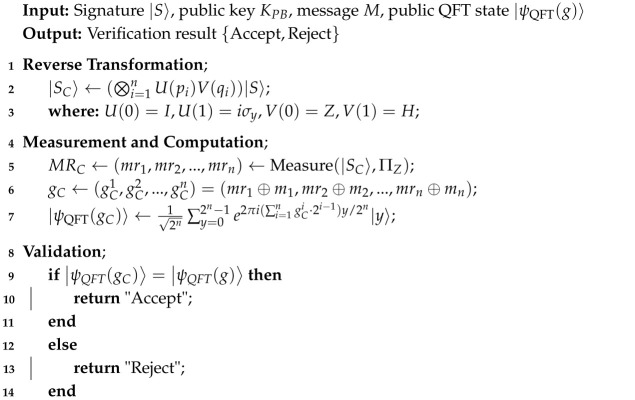


**Figure 1:**
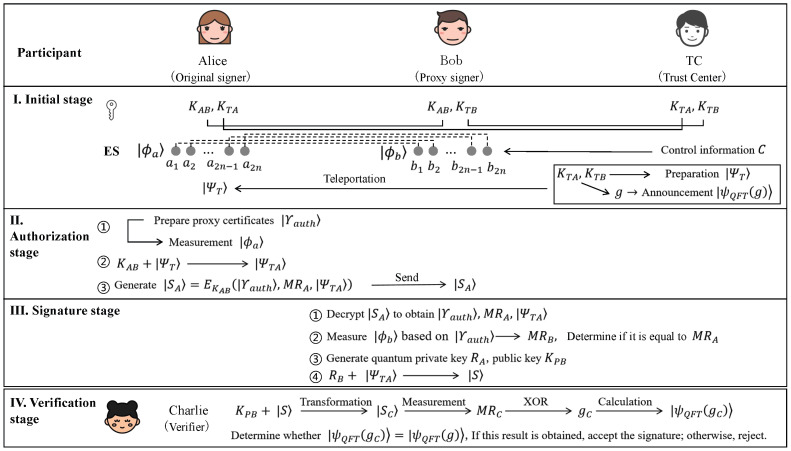
The schematic diagram of the proposed quantum proxy signature scheme.

The schematic diagrams of each stage of the scheme are shown in Figure 1. The final proxy signature generated can be used for public verification and can be carried out at any time after the signature stage is completed. There is no limit on the number of verifiers who complete the verification. Due to the openness of information, the verification information of the completed verifiers does not affect the verification of subsequent verifiers. Multiple verifiers can conduct the verification simultaneously, which has a high signature verification efficiency.

## 4. Security Analysis

The security of the proposed proxy signature scheme for individual original signatories is analyzed below. The security analysis follows the established framework common in digital signature schemes [40,41], which ensures a comprehensive evaluation by examining the following three critical aspects: anti-honesty termination, anti-repudiation attack, and anti-forgery attack.

### 4.1. Anti-Honesty Termination

The anti-trust termination property of the proposed scheme mainly indicates whether the verifier can successfully verify the signature when all participants are honest and there is no external attack. In the initial stage, the trusted center (TC) prepares the quantum state sequence $|\Psi_{T}\rangle$ based on the keys $K_{TC}$ and $K_{TB}$, and this sequence subsequently evolves into

(31) $$|\Psi_{T}\rangle \overset{K_{AB}}{\to }|\Psi_{TA}\rangle \overset{R_{B}}{\to }|S\rangle \overset{K_{PB}=R_{B}⨁K_{TB}⨁K_{AB}}{\to }|S_{C}\rangle .$$

Due to the unitary transformations performed during the evolution process, based on the Equations (11), (19), (22) and (23), the relationship can be obtained as follows

(32) $$MR_{i}=c_{i}⨁e_{i},$$

Therefore, it can always ensure

(33) $$g_{C}=g,\;|\psi_{\mathrm{QFT}}\left(g_{C}\right)\rangle =|\psi_{\mathrm{QFT}}\left(g\right)\rangle ,$$

That is, in a situation where there is no interference from either internal or external dishonesty, the signature can always be verified as successful.

### 4.2. Anti-Repudiation Attack

Firstly, for the original signer Alice, since the quantum states $|\Psi_{T}\rangle$, $|\psi_{\mathrm{QFT}}\left(g\right)\rangle$, $|\Psi_{TC}\rangle$ and the public key $K_{PB}$ involve the key $K_{AB}$ between Alice and Bob, as well as the key $K_{TC}$ between Alice and TC, if the final verification is successful, Alice’s use of the key makes it impossible for her to deny that she performed the proxy authorization. For the proxy signer Bob, since the proxy authorization certificate $|\Phi_{w}\rangle$ is a known quantum state containing Bob’s identity information, and the signature |S〉 and the public key $K_{PB}$ are derived from the shared key $K_{TB}$ and $K_{AB}$ held by Bob, thus Bob cannot deny that he performed the proxy signature on the message *M*.

### 4.3. Anti-Forgery Attack

The anti-forgery property of the proposed scheme can be analyzed from two aspects: anti-external attack and anti-internal attack.

For external attacks, assume the attacker is Eve, and her possible attack methods include auxiliary particle attack and interception retransmission attack. When Eve conducts an auxiliary particle attack, she uses the entangled pairs generated between Alice and Bob to entangle with them. Since the entangled pairs between Alice and Bob are generated by the given entanglement establishment process and not generated and distributed separately by TC, the scheme can effectively resist the auxiliary particle attack. If Eve chooses to carry out an interception retransmission attack, she needs to intercept the transmitted quantum state and replace it with a tampered quantum state. Since the transmission of quantum states in the given scheme uses encryption with the key generated by quantum key distribution, its security is guaranteed by the one-time key generated by quantum key distribution. When considering unconditional security for one-time pad encryption, the proposed scheme can completely resist this attack.

For the internal forgery attack, consider that both the original signer Alice and the proxy signer Bob could be the implementers of the forgery attack. If Alice is the attacker, she might forge the proxy signature $|S\rangle$ that should have been generated by Bob based on $|\Psi_{TA}\rangle$. The generation of signature $|S\rangle$ requires the participation of keys $K_{TB}$ and $K_{AB}$, as can be seen from Equation (24), $|S\rangle$ is obtained from $|\Psi_{TA}\rangle$ through a specific unitary transformation. If Alice forges $|S\rangle$ merely by guessing the key, the probability of success is

(34) $$P_{\mathrm{Forged}\;\mathrm{by}\;\mathrm{Alice}}=\frac{1}{2^{4n}}.$$

If Bob is the perpetrator of the forgery attack, he might carry out the forgery by generating an effective signature that is different from $|S\rangle$. According to the equation (14), the trusted center TC calculates and makes public the quantum state $|\psi_{QFT}\left(g\right)\rangle$ after the action of the one-way function based on quantum Fourier transform. This means that the message to be signed *M* cannot be forged or altered. According to the properties of the quantum one-way function, Bob cannot generate two different signatures using the same message. Moreover, the successful verification of the signature depends on the key $K_{TC}$ between Alice and TC, and Bob, without this key information, cannot forge other signatures that can be successfully verified.

## 5. Summary

This paper presents a quantum proxy signature scheme that allows any number of verifiers to validate the validity of the signature. This scheme can be used when any number of network nodes participate in the signature verification process, and there is no need for the verification nodes to have prior information exchange with the nodes involved in the signature. It has high efficiency and flexibility in network applications. The use of one-way functions based on quantum Fourier transformation in the signature relies on the principles of quantum state non-clonability and the difficulty of precisely controlling quantum entanglement, which are quantum characteristics. The cryptographic foundation of this scheme guarantees its security against future quantum attacks, meaning that even with highly developed quantum computers, it is infeasible for attackers to reverse the input of the function.

## Data Availability

The original contributions presented in this study are included in the article. Further inquiries can be directed to the corresponding author
